# The Longitudinal Aging Study Amsterdam: cohort update 2016 and major findings

**DOI:** 10.1007/s10654-016-0192-0

**Published:** 2016-08-20

**Authors:** Emiel O. Hoogendijk, Dorly J. H. Deeg, Jan Poppelaars, Marleen van der Horst, Marjolein I. Broese van Groenou, Hannie C. Comijs, H. Roeline W. Pasman, Natasja M. van Schoor, Bianca Suanet, Fleur Thomése, Theo G. van Tilburg, Marjolein Visser, Martijn Huisman

**Affiliations:** 1 Department of Epidemiology and Biostatistics, EMGO + Institute for Health and Care Research, VU University Medical Center, Amsterdam, The Netherlands; 2 Department of Sociology, VU University, Amsterdam, The Netherlands; 3 Department of Psychiatry, EMGO + Institute for Health and Care Research, VU University Medical Center, Amsterdam, The Netherlands; 4 Department of Public and Occupational Health, EMGO + Institute for Health and Care Research, VU University Medical Center, Amsterdam, The Netherlands; 5 Department of Health Sciences, Faculty of Earth and Life Sciences, EMGO + Institute for Health and Care Research, VU University, Amsterdam, The Netherlands; 6 Department of Internal Medicine, Nutrition and Dietetics, VU University Medical Center, Amsterdam, The Netherlands

**Keywords:** Longitudinal studies, Cohort studies, Netherlands, Epidemiology, Aging, Biomarkers, Health status indicators, Social support, Cognitive function, Mental health

## Abstract

The Longitudinal Aging Study Amsterdam (LASA) is an ongoing longitudinal study of older adults in the Netherlands, which started in 1992. LASA is focused on the determinants, trajectories and consequences of physical, cognitive, emotional and social functioning. The study is based on a nationally representative sample of older adults aged 55 years and over. The findings of the LASA study have been reported in over 450 publications so far (see www.lasa-vu.nl). In this article we describe the background and the design of the LASA study, and provide an update of the methods. In addition, we provide a summary of the major findings from the period 2011–2015.

## Introduction

The Longitudinal Aging Study Amsterdam (LASA) was initiated by the Dutch Ministry of Welfare, Health and Culture (currently Ministry of Health, Welfare and Sports) in the early 1990s in response to population aging in the Netherlands. Ministry officials recognized that demographic changes were leading to an increase in the proportion of older people in the Netherlands, and that these changes would shape future health care use. Multi-disciplinary and longitudinal observational research was considered to be needed to inform the ministry’s policy and to monitor functioning and well-being of Dutch older people. The LASA study was designed by researchers from the VU University and VU University Medical Center in Amsterdam, in a close collaboration between social and biomedical scientists. This collaboration ensured a thorough multi-disciplinary approach fitting the scope of the intended focus of LASA. The main objective of LASA was to study the determinants, trajectories and consequences of physical, cognitive, emotional and social functioning [1]. The study started in 1992 and is still ongoing. LASA is one of a few prospective studies of older adults in the Netherlands [2–4].

In this paper we describe the design of LASA, and provide an update of the methods. Furthermore, we provide an overview of the main findings that were obtained with LASA data from the past 5 years (2011–2015).

## The design of LASA

LASA is a prospective cohort study, initially based on a nationally representative sample of older adults aged 55–85 years (born between 1908 and 1937) from three regions in the Netherlands. These three regions (around Zwolle, Oss and Amsterdam) were selected to obtain an optimal representation of the Dutch older population, with respondents from the predominantly protestant northeast, the largely catholic south and from the more secularized western part of the Netherlands and from both urbanized and rural areas within these regions. The sample is used in two studies: first the NESTOR study on Living Arrangements and Social Networks (LSN) of older adults [5], and second LASA. The sample was randomly selected from municipal registries in 1992, with an oversampling of the oldest old and older men. The initial response rate (defined as the number of complete and partial interviews, divided by the total number of eligible persons in the sample plus a fraction of those persons who were in the sample but of whom eligibility could not be determined) was 60 % (n = 3805). The cooperation rate (defined as the number of completed interviews divided by the total number of contacted eligible persons) was 62 %.

**Fig. 1:**
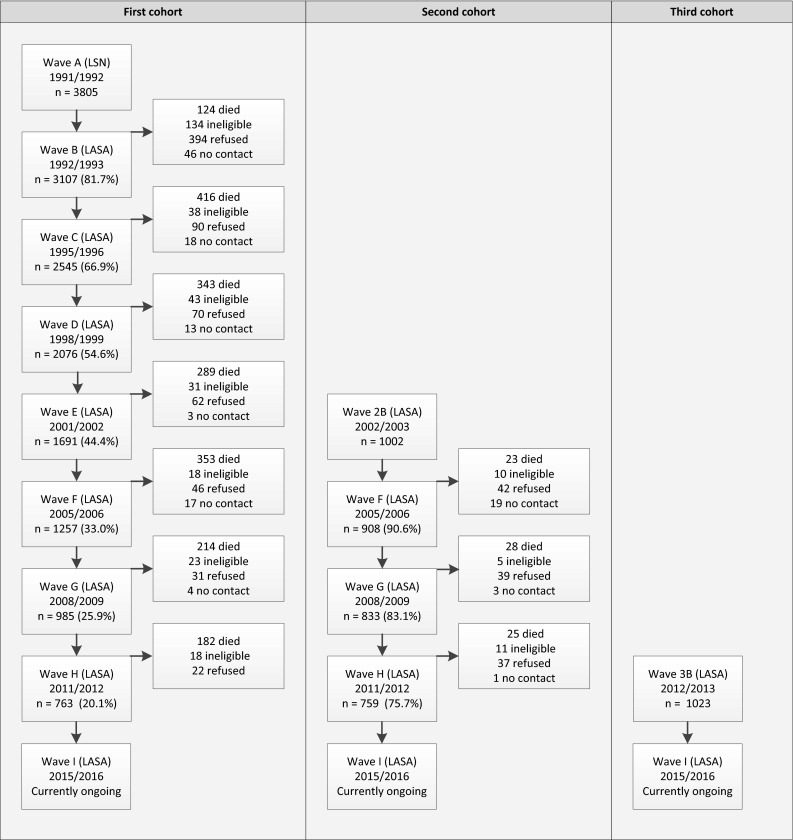
LASA study design, survival and participation

On average, 11 months after the LSN interview (wave A), participants were invited to participate in the first measurement wave of LASA (wave B), with a response rate of 85 % and a cooperation rate of 89 %. Since the 1992 LSN interview, there have been eight LASA measurement waves to date (Fig. 1). At the seventh measurement wave (wave H), approximately 20 years after the start of LASA, a total of 763 respondents of the original sample was retained.

Two additional cohorts of respondents were recruited from the same sampling frames exactly 10 and 20 years after the baseline measurement of the initial sample: one in 2002–2003, and another in 2012–2013. The realized sample size for the three cohorts is shown in Table 1. The second cohort that was included in 2002–2003 consisted of 1002 men and women born between 1938 and 1947 (cooperation rate was 62 %), and the third cohort from 2012 to 2013 consisted of 1023 men and women born between 1948 and 1957 (cooperation rate was 63 %). In subsequent measurement waves, respondents from these new cohorts were merged with those from the original cohort. In 2016, the data collection of the most recent LASA measurement wave (2015–2016, wave I) will be completed. This wave includes the remaining respondents from all three cohorts.

**Table 1:** Realized sample size for LASA cohort baseline measurements and wave H

Cohort	Birth years	1992–1993	2002–2003	2011–2012	2012–2013
		Wave B^a^	Wave 2B^a^	Wave H^a^	Wave 3B^a^
		Main interview/medical interview^b^	Main interview/medical interview	Main interview/medical interview	Main interview/medical interview
First cohort	1908–1912	580/460		6/1	
	1913–1917	575/476		33/15	
	1918–1922	472/412		62/40	
	1923–1927	492/432		159/100	
	1928–1932	512/468		240/182	
	1933–1937	476/423		263/212	
Second cohort	1938–1942	–	508/464	370/315	
	1943–1947	–	494/455	389/347	
Third cohort	1948–1952	–	–	–	523/460
	1953–1957	–	–	–	500/429
	Total	3107/2671	1002/919	1522/1212	1023/889

^a^For all other measurement waves, realized sample size by cohort are shown in Fig. 1 and details on realized sample size according to year of birth are published elsewhere [1]

^b^All participants in the medical interview also participated in the main interview

The attrition of respondents over time is a specific concern of longitudinal studies on aging. For this reason men and the oldest old were initially oversampled to ensure that there would be reasonable numbers of very old men, even after many years of follow-up. Attrition in the first LASA cohort can be mainly attributed to mortality (Fig. 1), and for a relatively small proportion to frailty of respondents and refusal to cooperate. Details on the attrition in LASA, and the associations with participant characteristics have been published earlier [1, 6].

The LASA study is conducted in line with the Declaration of Helsinki, and was approved by the medical ethics committee of the VU University medical center.

## Methods update

### Measurements

Data are collected by trained interviewers who visit the respondents at home. Each measurement wave has three components: a main interview, a self-report questionnaire, and a medical interview. The main interview takes, on average, almost 2 h to complete. To obtain additional data, respondents are asked to fill out a written questionnaire separately. This questionnaire is left at the respondent`s home during the visit for the main interview, and is collected during the medical interview or returned by mail. As of 2015, respondents also have the option to complete this questionnaire online. Respondents are then asked to participate in a subsequent medical interview. After consent, a separate visit is scheduled in which clinical measurements are administered and additional questions are asked. The medical interview takes on average 1 h and a half to complete. Diagnostic psychiatric interviews are administered subsequently to respondents who scored highly on the symptom checklists of depression or anxiety during the main interview.

The data collection includes measures for each of the four functioning domains that are central in the study: physical, cognitive, emotional and social functioning. The main predictors and outcome measures are shown in Table 2. For reasons of longitudinal comparison, measurement instruments of LASA mostly remain the same across measurement waves. Nevertheless, sometimes measurements are updated or improved (e.g., the original dynamometers to measure grip strength have been replaced by new ones in 2011–2012, and the questionnaire on internet and computer use has been updated in 2011–2012 due to rapid developments in ICT), new measurements are added to the data collection, and old ones are removed. New measurements added in the past 5 years include cognitive tests for executive functioning (word fluency, letter fluency and digit span) from measurement wave H on, a short questionnaire based on type and frequency of music making activity (from wave H on), Parkinson assessment (wave 3B), spirometry (wave 3B), the Perceived Stress Scale (wave H and 3B) [7], food security and risk of malnutrition (from wave H on), physical and psychosocial work demands and psychosocial resources at work (from wave H on), use of help with nursing tasks, transport and arrangement of care, and perceived control of care (from wave H on), vitamin supplement use (wave I), oral health and oral care questionnaire (wave I), and objective assessment of physical activity and sedentary behavior by accelerometry (wave I).

**Table 2:** Main predictors and outcome measures in LASA

Measure	Details	Measurement waves									
*Physical functioning*											
Body composition	Anthropometry	B	C	D	E	2B	F	G	H	3B	I
Lifestyle factors	Self-report	B	C	D	E	2B	F	G	H	3B	I
Chronic diseases	Self-report	B	C	D	E	2B	F	G	H	3B	I
Blood pressure	Blood pressure monitor	B	C	D	E	2B	F	G	H	3B	I
Functional limitations	Self-report	B	C	D	E	2B	F	G	H	3B	I
Physical performance	Performance tests	B	C	D	E	2B	F	G	H	3B	I
Pain	Self-report	B	C	D	E	2B	F	G	H	3B	I
Falls/fractures	Self-report	B	C	D	E	2B	F	G	H	3B	I
Medication	ATC codes	B	C	D	E	2B	F	G	H	3B	I
Self-rated health	Self-report	B	C	D	E	2B	F	G	H	3B	I
*Cognitive functioning*											
General cognitive functioning	MMSE, coding task	B	C	D	E	2B	F	G	H	3B	I
Intelligence fluid	RCPM	B	C	D	E	2B	F	G			
Intelligence crystallized	GIT		C				F				I
Executive functioning	Verbal fluency, digit span								H	3B	I
Memory: everyday memory, memory complaints	15WT, self-reported complaints	B	C	D	E	2B	F	G	H	3B	I
*Emotional functioning*											
Anxiety	HADS-A	B	C	D	E		F	G	H	3B	I
Depression^a^	CES-D	B	C	D	E	2B	F	G	H	3B	I
Life events	List of life events		C	D	E		F	G	H		I
Personality traits	Scales for self-esteem, mastery and self-efficacy	B	C	D	E	2B	F	G	H	3B	I
	Neuroticism scale	B	C	D	E	2B				3B	
Sleep quality	Self-report	B	C	D	E	2B	F	G	H	3B	I
Quality of life	EuroQol				E		F	G	H	3B	I
*Social functioning*											
Personal network: size, social support	Self-report	B	C	D	E	2B	F	G	H	3B	I
Loneliness	De Jong Gierveld loneliness scale	B	C	D	E	2B	F	G	H	3B	I
Social participation	Self-report	B	C	D	E	2B	F	G	H	3B	I
*Other*											
Demographic and socio-economic factors	Self-report	B	C	D	E	2B	F	G	H	3B	I
Religion, religiosity	Self-report		C	D	E	2B	F	G	H	3B	I
Use of care	Self-report	B	C	D	E	2B	F	G	H	3B	I
Biomaterial measurements: see Table 3											

See the LASA website (www.lasa-vu.nl) for a complete overview and details of all study measures

Abbreviations: *ATC* anatomical therapeutic chemical classification system, *MMSE* mini mental state examination, *RCPM* raven coloured progressive matrices. *GIT* Groninger Intelligence Test, *15WT* 15 Word Test, *HADS-A* Hospital Anxiety Depression Scale-Anxiety subscale, *CES-D* Center for Epidemiologic Studies Depression scale

^a^DSM-IV diagnoses are available for a subsample

### Biomaterial measurements

Table 3 provides an overview of the realized sample sizes for biomaterial measurements that were assessed throughout the course of the LASA study. Morning blood samples have been collected in 1992–1993 (wave B: the first cohort, only for those living in the Zwolle and Amsterdam region), 1995–1996 (wave C: the first cohort), 2002–2003 (wave 2B: the second cohort), 2008–2009 (wave G: the first and second cohort) and 2012–2013 (wave 3B: the third cohort). Currently, information on a multitude of biomarkers is available for the first and the second cohort. New blood determinations may become available in the future, when new measurements are done for specific research questions. Serum, EDTA, and aprotinin samples are available, as well as DNA.

**Table 3:**
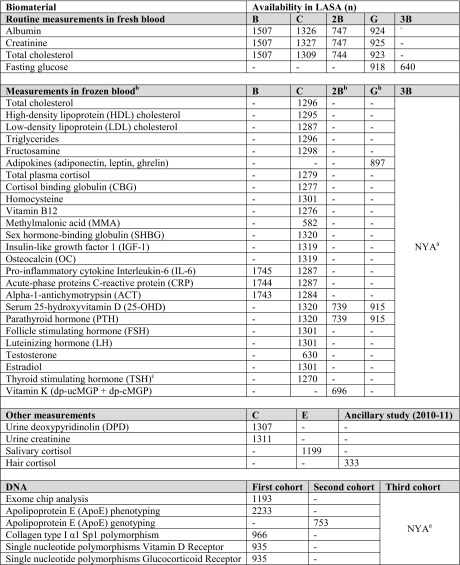
Realized sample size for biomaterial measurements in LASA

^a^Not yet available: blood has been collected (n = 640), but assessments have not yet been performed

^b^New blood determinations may become available in the future from frozen blood

^c^In case of abnormalities in TSH level, Tri-jodothyronin (T3) and/or Thyroxin (T4) were measured

### Follow-up procedures and incidental findings

LASA respondents are visited approximately every 3 to 4 years in their homes (Fig. 1). Mortality status is retrieved approximately every 2 years from registers of the municipalities where respondents are living. At this moment, mortality data is available until July 1, 2015. In 1995, 2000, 2005, and 2010 data on chronic diseases were obtained from general practitioner records and compared with self-reported data [8, 9]. Upon request, and with strict regulations, data from LASA can be linked with data of Statistics Netherlands (e.g., to determine cause of death or healthcare use).

The procedure with regard to incidental findings is as follows: in general, no individual feedback is provided to respondents. However, for some clinical measurements results are provided to the general practitioner, but only with consent from the respondent. This concerns the routine measurements in fresh blood (albumin, creatinine, total cholesterol and fasting glucose), high or low blood pressure, and extreme values found at the examination of blood samples from frozen blood.

### Ancillary and integrative studies

Several ancillary studies have been performed among subsamples of LASA participants to enrich the data from regular measurement waves, with the purpose to answer specific research questions. For example, in the context of the European Project on Osteoarthritis (EPOSA) [10, 11], additional data on osteoarthritis and joint pain have been collected in 2010–2011 and 2015–2016 among initially 574 LASA respondents. Other ancillary studies focused on various issues, such as end of life, family caregiving to older adults, perceived control in healthcare, nutrition and food-related behavior, loneliness behavior, Attention-Deficit/Hyperactivity Disorder, and predictors of hearing help-seeking behavior.

Reflecting developments in epidemiological research, data from LASA are increasingly being used for integrative studies, such as meta-analyses and international collaborations. In the past years, various meta-analyses have been performed including LASA data on mortality, physical functioning and biomarkers (e.g., [12–19]). We also participate in the Integrative Analysis of Longitudinal Studies on Aging (IALSA) [20, 21], which has the aim to evaluate the reproducibility of results from longitudinal and life course studies, predominantly in the domains of cognitive and emotional functioning.

## Major findings

Over 450 scientific publications have been published based on LASA data, of which at least 150 have been published in the past 5 years (2011–2015). These publications cover a broad range of topics, and many studies address associations across domains of functioning. A full list of publications can be found on the LASA website (www.lasa-vu.nl). Research with LASA data covered topics in public health research, clinical epidemiology, psychiatric epidemiology, nutritional epidemiology and social epidemiology. Here, we present major findings from the past 5 years according to important LASA research themes: time trends in health, biomarkers, vitamin D, nutrition, lifestyle, frailty, sensory functioning, depression, Attention-Deficit/Hyperactivity Disorder, cognitive decline, personal networks, loneliness, employment and retirement, care, and end of life.

### Time trends in health

One major health trend in the past decades is characterized by the increase in life expectancy at older ages. Much debate revolves around the question if the added life years are healthy or unhealthy years, as the answer affects projected health care costs [22, 23]. LASA data showed that disease-specific 5-year mortality declined substantially from 1998 to 2008 in 78–87-year-olds in the Netherlands, especially for diabetes, heart disease and cancer [24]. This was attributed in part to the rise in educational level and in part to the improved treatment of disease, which allows older people to live longer with their diseases.

To what extent do these additional years with disease lead to disability? A meta-analysis in five population-based surveys, spanning the years 1990–2007, showed stable trends for limitations in 10 out of 12 daily activities. Moderate, but not severe, limitations increased for two activities [25]. Also chronic disease prevalence increased over time [26]. The disabling effect of chronic diseases on functioning depended on the measurement instrument used for functional limitations: no change was seen for items from the Organisation for Economic Cooperation and Development scale, but for items from the Short Form Health Survey-36, the disabling effect slightly decreased [26].

A further question is if older people themselves view their health as better or worse over time. In LASA-participants, self-rated health remained stable over 17 years [27]. However, the objective information upon which older people based their self-ratings appeared to have changed over time. The association of self-rated health with chronic diseases became weaker, but its association with severe disability became stronger. Suggested explanations were earlier-stage diagnosis of disease, improved disease management and survival, and delay of severe (but not mild) disability amongst others by increased availability of assistive technology. Also, increased over reporting of chronic diseases might have been a factor [8].

The onset of a disease is an experience outside the control of the patient, and thus may be detrimental to their sense of mastery, especially in midlife when disease onset is still relatively rare. Over a 3-year period, mastery declined in 55–64-year-olds who had a chronic disease, both in an earlier (observed 1992–1996) and more recent cohort (observed 2002–2006) [28]. In heart disease patients in the recent cohort, however, mastery improved over a 3-year period. This intriguing finding might be attributed to the great improvement in treatment of heart disease during the study period, allowing heart patients to lead normal lives.

Individual strategies to cope with disability included the adoption of assistive technology (AT). Between 1992 and 2006, AT-use increased substantially [29]. Despite its beneficial effects, AT may be stigmatising and thus affect self-rated health negatively. Given the same level of disability, AT-users rated their health as worse compared to non-users. However, longitudinal data showed that this was only true for people who had recently started AT-use.

### Biomarkers

The concentrations of many biomarkers change with aging, which may have negative health consequences, even when the changes are within the normal range. Serum insulin-like growth factor 1 (IGF-1) levels decrease during aging. The association of IGF-1 concentration with (components of) the metabolic syndrome was examined in LASA respondents. It was observed that high-normal IGF-1 levels were associated with the metabolic syndrome and high triglyceride levels [30]. Furthermore, the role of the metabolic syndrome in the association between IGF-1 and incident cardiovascular disease (CVD) was studied. Respondents with the metabolic syndrome and high IGF-1 levels had an increased risk of CVD [30]. LASA data were also included in a study using a family design. It was shown that higher serum IGF-1 levels in middle age were associated with risk of Alzheimer’s disease in older age, independent of Apolipoprotein E (ApoE) genotype [31].

Previous studies observed an association between elevated homocysteine levels and fractures in LASA data [32]. As decreased physical performance may precede the fracture risk, the association between elevated homocysteine levels and physical performance was examined. It was found that women in the highest quartile of homocysteine had a significantly lower physical performance than women in the lowest quartile [33]. Moreover, higher homocysteine levels were associated with lower grip strength and more functional limitations in men [34]. In women, higher homocysteine levels were associated with more functional limitations after 3 years [34]. No associations were observed with muscle mass or falling [34]. Finally, an increased risk of nursing home admission and mortality in older women was observed, but not in older men with higher levels of homocysteine [35].

Several studies used LASA data to examine cortisol levels in relation to negative health outcomes. In a meta-analysis using individual participant data, more dynamic activity of the hypothalamic–pituitary–adrenal (HPA) axis, i.e. greater decline in diurnal cortisol release, was associated with better physical performance [14]. Lower morning cortisol levels, higher evening cortisol levels, and flattened diurnal variability of cortisol levels were associated with increased risk for memory decline in ApoE-ε4 carriers but not in non-carriers [36]. In addition to blood and salivary cortisol, long-term cortisol levels were assessed in 3-cm hair segments. High hair cortisol levels were cross-sectionally associated with CVD and type 2 diabetes mellitus [37].

It was shown that persons with a serum Thyroid stimulating hormone (TSH) in the upper quartile had a higher prevalence of the metabolic syndrome as compared with persons in the lowest quartile [38]. Free thyroxin (T4), but not TSH, was associated with erythrocyte indices [39].

Because LASA data are obtained from a representative sample of the Dutch older population, the data can be used to establish national reference values. Data from LASA and the Netherlands Study of Depression and Anxiety (NESDA) were used to calculate assay specific parathyroid hormone (PTH) reference levels in relation to 25-hydroxyvitamin D (25-OHD) levels [40]. In this study, PTH reference values differed by 25-OHD status, showing the importance of establishing PTH reference values in a local reference population taking 25-OHD status into account. Another study examined the cross-sectional association between PTH and CVD. Persons in the highest quintile of PTH had significantly higher odds of CVD as compared with persons in the lowest quintile [41].

Finally, LASA data were included in several large genetic meta-analyses and replication studies. Most of these studies examined genetic variations for osteoporosis and fractures [15–17, 42–45].

### Vitamin D

In LASA respondents, long-term serum 25-OHD levels remained fairly stable during aging with slightly increasing levels in persons aged 55–65 years and slightly decreasing levels in persons aged 65–88 years [46]. The main function of vitamin D concerns calcium homeostasis. As previous studies have been inconsistent with regard to the association between serum 25-OHD, bone mineral density (BMD) and quantitative ultrasound, it was explored whether these associations were modified by body mass index (BMI), age, gender, or physical activity [47]. In respondents aged 65 years and older from the first LASA cohort and in the ‘B-vitamins for the prevention of osteoporotic fractures’ (B-PROOF) study, it was found that only in case of low-to-normal BMI (<25 kg/m^2^), serum 25-OHD < 25 nmol/L was associated with lower Broadband Ultrasound Attenuation (BUA) values as compared to the reference group (≥50 nmol/L) [47]. These individuals also had lower BMD at the hip and lumbar spine [47]. In respondents aged 55–65 years from the second LASA cohort, no associations with BUA were observed [47]. BMD was not assessed in these respondents.

As the vitamin D receptor is expressed in virtually every human tissue, this suggests a more widespread function than calcium homeostasis alone [48]. In LASA respondents, the association of serum 25-OHD has been examined with several outcomes. Serum 25-OHD was associated with physical performance, as assessed by the walking test, chair stands test and tandem stand, in persons aged 65 years and older [49]. In persons aged 55–65 years, an association with physical performance was observed, but not with decline in physical performance [49]. Furthermore, we found that a low serum 25-OHD was associated with functional limitations [50], the metabolic syndrome [51], peak expiratory flow rate in men [52], lower quality of life and self-rated health [53].

The threshold for a sufficient serum 25-OHD concentration is still under debate and may vary according to outcome and subgroup. Spline curves were used to estimate thresholds for falling, fractures, hypertension, cardiovascular disease, blood pressure, PTH, grip strength, physical performance, functional limitations, BMI, and mortality [54]. The estimated thresholds differed by sex and age groups, and varied from 46 (PTH) to 68 nmol/L (hypertension) [54].

Finally, two models with easy assessable variables were developed to predict serum 25-OHD concentrations below 50 nmol/L and below 30 nmol/L, respectively [55].

### Nutrition

In the Lifestyle ancillary study, information on fruit, vegetable and fish consumption was assessed in 1058 older adults, as well as behavioral information regarding food consumption, including motivation to eat healthy, perception of intake, and barriers for meeting the Dutch recommendation for fruit, vegetable and fish consumption. The most reported motivations to eat healthily were feeling fit, current health and body weight [56]. Based on the self-reported adherence to the Dutch dietary recommendations, data showed that almost one out of five older adults overestimated their intake of vegetables [57]. Clear intake differences between education and income groups were also observed, with older adults with a lower socioeconomic status having a more unhealthy diet [58]. Lower socioeconomic groups perceived more barriers for meeting the dietary recommendations, such as the high price of fish [59].

Obesity and undernutrition are two important malnutrition problems in older adults. Of all age groups, obesity is most prevalent among the older population of the Netherlands. Nowadays, a high waist circumference (>102 cm for men and >88 cm for women) is considered a valuable indicator of overweight. However, LASA data suggest that these cut points are too strict for older persons, as they are not optimal for predicting disease risk [12, 60]. Especially for women the cut point should be increased. Recent research also showed that only about 10 % of obese older men and women perceived their body weight status correctly and considered themselves obese [61].

Underweight and weight loss frequently occur in old age [62] and are markers of undernutrition, which is associated with poor health outcomes and early mortality. A poor appetite is a strong determinant of undernutrition of older persons [63]. Not all weight loss appeared to be detrimental: the association differs according to self-reported reasons of weight loss [64]. Recent involuntary weight loss due to medical reasons or unknown reasons increased mortality risk, while involuntary weight loss due to social reasons and voluntary weight loss (due to energy-restricted diet and/or increased physical activity) did not increase this risk. LASA data also showed that a low mid-upper arm circumference in older adults is a reproducible measure [65] and a more sensitive measure of underweight compared to a low BMI [65]. This measure has been incorporated in a new malnutrition screening tool for community-dwelling older adults, the SNAQ65+, which was developed using LASA data [66].

Recently, more extensive dietary information has been collected in 1430 LASA participants using a 238-item food-frequency questionnaire and an additional questionnaire focusing on dietary habits, including meal preparation and food shopping, emotional eating, meal pattern and appetite. In addition, to complement these data, three 24-h recalls were obtained in a random sample of 93 participants. These new dietary data are now being analyzed in ongoing research.

### Lifestyle

Physical activity is an important determinant of healthy aging. Unfortunately, based on objective measurement of physical activity using accelerometry in a random sample of LASA participants, only 25 % adhered to the Dutch recommendation for physical activity [67]. However, based on self-report and after explaining what the Dutch recommendation is, 57 % of these participants indicated that they were meeting the recommendation, showing a large overestimation of current physical activity by older adults [67]. This overestimation will likely reduce the impact of public health messages to increase physical activity.

Sedentary behavior is defined as the time spent on activities with very low energy expenditure during the waking hours, i.e. sitting and lying down. A questionnaire to assess sedentary behavior in older adults was developed and validated against accelerometry [68]. Six questions about daily sedentary activities were sufficient to rank older adults with regard to sedentary activity level (h/day). The questionnaire was completed by 1278 participants of wave G (2008–2009) and showed that older adults spend an average of 10.3 h/day being sedentary. More importantly, compared to those who were the least sedentary (lowest 25 %), those who were the most sedentary (highest 25 %) experienced a greater decline in physical functioning during the subsequent 3 years, and were more likely to die in the subsequent 6 years [69]. Currently, objective measurements of physical activity and sedentary behavior on seven consecutive days are being obtained from LASA participants using the Actigraph GT3X accelerometer (2015–2016, wave I).

LASA data indicate a clear time trend across generations of decreasing prevalence rates of current smoking in older men but not (yet) in older women [70, 71]. Older men and women who continue to smoke into old age were often not satisfied with their smoking behavior and would like to stop [71]. The prevalence of excessive alcohol consumption increased across generations of respondents. Heavy alcohol use (>3 glasses per day for men and >2 glasses per day for women) was reported by 13.4 % of the LASA participants [72].

### Frailty

The concept of frailty is widely used in geriatrics. Frailty is usually defined as the loss of reserve capacity in one or more domains of functioning, which increases the risk of adverse health outcomes, such as hospital admissions and mortality [73, 74]. The loss of reserve capacity may be only physical or in multiple domains, including the cognitive and psychosocial domain.

A well-known operational definition of physical frailty is the frailty phenotype based on the presence of at least three of the following five criteria: weight loss, weak grip strength, exhaustion, slow gait, and low physical activity [75]. Using the frailty phenotype, LASA data have been used to study the association between educational level and frailty in later life [76]. Older adults with a lower educational level were more often frail compared to those with a higher educational level, and these differences remained during 13 years of follow-up. Educational differences in frailty were for a large part explained by differences in material, biomedical, behavioral and mental factors [76]. Adverse effects of the frailty phenotype on social functioning were also assessed. Physical frailty was associated with a smaller network size and higher levels of loneliness, and with an increase in loneliness over a period of 3 years [77]. Additionally, potential effect modification by psychosocial resources of the effects of physical frailty on functional decline and mortality was assessed. Frailty was associated with functional decline and mortality, but there was no evidence that psychosocial resources such as mastery and social support buffered against the adverse health outcomes of frailty [78].

Broader definitions of frailty, including cognitive and psychosocial markers, have also been operationalized with LASA data. When using such a broad definition, associations between frailty and falls and fractures were found [79]. However, frailty was not a better predictor of recurrent falls than falls history [79]. In 2011, the Tilburg Frailty Indicator, a multidimensional frailty instrument, was constructed with LASA data to describe longitudinal trajectories in frailty and subdomains of frailty: physical, social and psychological frailty. There was a large variability in frailty trajectories in older adults, suggesting that frailty is dynamic [80].

Not much is known about the relative importance of frailty components. Data from fives studies, including LASA, were analyzed to investigate the association between seven frailty domains (nutrition, physical activity, mobility, strength, energy, cognition and mood) and their relative contribution in explaining differences among individuals [81]. The frailty components aggregated consistently in the five study samples, suggesting a common underlying construct. Of the included frailty components, strength was the most discriminative characteristic [81].

### Sensory functioning

LASA data have been used to investigate the impact of hearing loss in great detail, based on both self-reported hearing status and a speech-in-noise test. A 7-year longitudinal study showed that older persons` ability to recognize speech in noise deteriorated significantly over time and that the rate of decline was roughly twice as large (i.e. about 0.26 dB signal-to-noise ratio per year) for those above the age of 75 years as compared to persons below this age (0.13 dB per year) [82]. In addition, decline in information-processing speed explained a moderate proportion of the decline in hearing [82]. Longitudinal associations between poor hearing and psychosocial health were investigated. Worse baseline hearing status, assessed by both the self-reported and objective hearing measures, was associated with loneliness at 4-year follow-up, but only in specific subgroups of older persons (e.g., men). No associations were observed with depressive symptoms [83]. The association of changes in hearing status with changes in psychosocial health was also investigated. Faster decrease in speech-in-noise recognition was associated with higher increase in emotional and social loneliness, but only for widow(er)s and persons with an already impaired hearing status. Again, no associations were found with depression [84].

In a cross-sectional study among 173 visually impaired older people, the LASA sample was used as a reference population. This study showed that levels of participation were lower among older adults with vision loss compared with the general older population [85]. Also, the prevalence of loneliness was higher among visually impaired older adults [86].

LASA data include information on the experience of dizziness. In a 7- and 10-year follow-up study, predictors of dizziness were investigated. Multiple factors predicted dizziness in older people, including living alone, having a history of dizziness, a history of rheumatoid arthritis or osteoarthritis, taking nitrates, the presence of anxiety or depression, impaired vision, and impaired function of the lower extremities [87].

### Depression

In LASA, subthreshold depression (SUBD) and major depressive disorder (MDD) are identified with a two-stage-screening procedure, including a symptom rating scale and diagnostic interview. In SUBD someone does not meet strict diagnostic criteria for MDD, but does have a clinically relevant level of depressive symptoms that seriously influence quality of life and functioning. SUBD in later life is common and important as prodromal state and prominent risk factor in the development of MDD. Incident SUBD occurred in 24 % of the participants [88]. A 17-year follow-up study on SUBD in later life showed a MDD incidence rate of 15.1/1000 person-years, which is twice as high compared to the general older population [89].

Several studies focused on risk factors for depression, interactions with comorbidity and outcomes of depression. Gait speed at baseline was significantly associated with incident SUBD in men, but not in women [88]. IGF-1 did not play an important role in the development of depression over time [90]. Low social support and a high need for social affiliation were associated with depression in later life, especially in men [91]. Higher levels of pain were associated with incident SUBD, whereas SUBD was not independently associated with the onset of pain [92]. Another study showed that the longitudinal association between pain and SUBD over time was largely explained by cognitive and psychological characteristics, such as mastery and neuroticism [93].

In persons without preexistent cardiac disease, depression was associated with future stroke but only in persons with low levels of neuroticism [94]. An unfavorable course of SUBD was found to be associated with smaller network sizes and higher levels of loneliness over time, especially in men and older participants [95]. Depression was also associated with non-psychiatric hospitalization, longer length of stay and higher mortality in clinical settings [96].

An association between religiousness and late-life depression was observed. Late-life depression seems to maintain a pervasive relationship over time with affective aspects of religiousness, such as having fear of God, feeling wronged by God, and negative religious coping [97]. Among those with a history of SUBD, church-membership, worship attendance and salience of religion were associated with a greater likelihood of depression in the last week of life [98], whereas among those without a history of SUBD aspects of religiousness were associated with a higher sense of peace [98].

Knowledge on adequate treatment of late-life depression and awareness of the negative consequences of long-term benzodiazepine use increased. However, overall benzodiazepine use remained stable between 1992 and 2002 among the Dutch population aged 55–64, with a high proportion of long-term users, despite the effort to reduce benzodiazepine use and the renewal of the guidelines in that period [99].

### Attention-deficit/hyperactivity disorder

Little was known about the prevalence and consequences of Attention-deficit/hyperactivity disorder (ADHD) in older adults. Therefore we included an ADHD screening list [100, 101] in wave G (2008–2009) as part of the medical interview. To subsequently diagnose ADHD, the Diagnostic Interview for ADHD in Adults, second edition (DIVA 2.0 [102]), which is based on DSM-IV TR criteria, was modified to a structured interview for use in LASA. All participants who scored high on the screener and a random sample of the participants in the moderate and low scoring group were approached for a diagnostic interview (n = 271).

The prevalence rate for ADHD in LASA respondents was 2.8 % [103]. Compared to older adults without ADHD, older adults with ADHD had a three times greater odds of being divorced or never married and reported more emotional loneliness [104]. They were also found to have lower self-esteem, lower self-efficacy, lower sense of mastery and higher levels of neuroticism and social inadequacy [105], and had higher levels of comorbid anxiety and depressive symptoms [106]. The increased risk of depression in older adults with ADHD was partly explained by being frequently involved in serious conflicts [107]. We also found an association between ADHD and poor cognitive performance, however, this association was largely explained by depressive symptoms [108].

The number of ADHD symptoms was positively associated with the presence of chronic non-specific lung diseases, cardiovascular diseases and the number of chronic diseases, and negatively with self-perceived health. There was no association between ADHD and lifestyle variables, such as smoking or alcohol use in this sample of older adults [109].

An additional, qualitative study among seventeen older adults who met the diagnostic criteria showed that the ADHD symptoms seemed to have had a more negative impact in the younger years than in the current lives of the respondents [110].

### Cognitive decline

The variability in cognitive decline among persons is high, which makes it difficult to distinguish whether decline in cognitive performance is due to normal cognitive aging or to a developing dementia. Therefore studies examined aspects of cognitive aging as well as risk factors for pathological cognitive decline. A decline in information-processing speed has long been considered a key driver of cognitive aging [111]. LASA data provide the opportunity to assess information-processing speed within persons over a long period of time. Results indeed showed that increasing age was related to lower information-processing speed, which in turn was associated with lower performance across repeated measures in other cognitive domains [111]. Several psychosocial factors were associated with cognitive performance or the rate of cognitive decline. For instance, frequent emotional support was associated with reduced feelings of loneliness and subsequently to better cognitive functioning. Increases in emotional support also directly enhanced cognitive performance [112]; this association was strongest amongst adults aged 65 years and older [112]. Furthermore, a reduction in social network complexity (a more homogeneous network including similar relationship types) was associated with poorer cognitive performance but not with the rate of decline in performance [113].

Highly stressful life events were associated with a higher rate of cognitive decline, whereas mild chronic stressors seemed to stimulate cognitive performance [114]. Persons who have experienced adverse childhood events showed a faster decline in information processing speed over a period of 10 years, but only in the presence of depressive symptoms. These data suggest that childhood adversity may cause a biological or psychological vulnerability, which is associated with both depressive symptoms and cognitive decline in later life [115]. A further study tried to disentangle the reciprocal associations between depressive symptoms and cognitive performance over 13 years of follow-up. Depressive symptoms in older patients were strongly associated with an increased likelihood of cognitive decline. On the other hand, older persons with slowed information processing speed were more vulnerable for an increase of depressive symptoms [116, 117].

HPA-axis dysregulation was associated with increased risk for memory decline in ApoE-ε4 carriers but not in non-carriers. This suggests that ApoE-ε4 carriers may be more vulnerable to the potential detrimental effect of HPA-axis dysfunction on verbal memory performance than non-carriers [36].

Studies on biomarkers of cognitive decline using LASA data in the last few years focused mainly on cholesterol and extracerebral cholesterol homeostasis. Independent associations were observed between high HDL cholesterol and better memory performance [118]. In addition, low LDL cholesterol was associated with worse general cognitive performance and faster decline on information processing speed. Inflammation markers (C-reactive protein, α-1-antichymotrypsin) appeared to mediate this association [118]. Extracerebral cholesterol homeostasis was associated with cognitive performance, but only in ApoE-ε4 non-carriers [119].

Memory complaints may be a precursor of measurable cognitive decline. Respondents who reported memory complaints more often showed impaired delayed recall and clinically relevant decline in learning ability [141]. For the early identification of persons at risk of memory complaints we developed classification models using a broad range of characteristics that are easy to assess in clinical practice, such as the use of medication, smoking history, hearing problems, sense of mastery and the presence of pain [120].

### Personal networks

The personal network is defined as people in different domains (e.g., children, neighbors) with whom there is frequent contact and who are considered to be important to the respondent [121]. The longitudinal nature of the LASA data and the inclusion of new cohorts of 55–64 year olds in 2002 and 2012 increased possibilities to study dynamics of social functioning and their impact on health.

Family relationships are known to be vital to older adults, as these provide emotional and instrumental support. Studies on the grandparent-grandchild relationship showed that adult grandchildren with a more intense relationship in childhood were more likely to be in the grandparents personal network [122], and that the importance of grandparental investment for receiving support from children depends on gender and the type of earlier investment. Grandparents who frequently provided child care for sons in the past more often received instrumental and emotional support from these sons [123]. Stepparents were more likely to include adult stepchildren in their personal network in recent times: the percentage of stepparents that had stepchildren in the network increased from 63 % in 1992 to 85 % in 2009 [124].

Younger generations of older people have networks that are more non-kin based. In younger generations, older adults were more likely to have friends and retain them longer in their personal network [125]. Older adults from younger generations (born after 1922) also have more non-kin in their networks and keep these well past the age of 75 [126]. It has also been assessed how the personal network changed after a move. Contextual conditions were found to be more important for starting relationships with new neighbors than personal conditions. In particular, those who moved to rural areas and felt safe, and those who moved to areas with lower priced homes, started new relationships with neighbors [127].

Among other things, research on personal networks is relevant to social epidemiology, because the network may be associated with health and mortality in old age. The network size declines with aging, due to a lack of replacement of lost relationships. In particular, persons with cognitive and mental health problems were at risk of declining network size [128]. It was investigated whether the associations with or pathways to mortality risk differ by characteristics of the network. Mortality risk was found to be lowest for older adults embedded in large and diverse networks [129].

### Loneliness

Loneliness is the subjective, unpleasant experience of a perceived discrepancy between the relationships one wishes and the quantity and quality of existing relationships (e.g., with spouse, children, friends) [130, 131]. Emotional loneliness occurs when an individual lacks a reliable attachment figure, like a partner. Social loneliness refers to the absence of a broader category of personal relationships such as extended kin, acquaintances, colleagues, and friends.

In data from recent LASA measurement waves, emotional loneliness was shown to increase the likelihood of institutionalization, especially among men [132]. The presence of active neighbor relationships appeared to be an effect modifier of this association and it buffered against the risk of institutionalization among the loneliest respondents [132]. Loneliness was also studied in relation to divorce, physical frailty, visual impairment, depression and cognitive functioning [77, 83, 86, 95, 133].

Awareness of risk factors for loneliness was assessed in an ancillary study adopting a vignette approach [134]. The results indicated that awareness of loneliness-provoking factors was high among respondents, in particular among those who were lonely themselves. For coping with loneliness, respondents aged between 62 and 100 suggested both active (improve relationships) and regulative (lower expectations) strategies [135]. Respondents’ own loneliness over 3 years was not associated with subsequent suggestions for active coping. Persistently lonely and recently recovered respondents endorsed lowering expectations more often than non-lonely and recently lonely respondents did [136].

### Employment and retirement

In order to attain the goal set by the Dutch government of increasing retirement age to 67 years by 2021, more insight is needed in ways to extend working lives. As evidence shows that health trends are not improving, however, working longer and maintaining adequate health may be at odds [137].

Recent research with LASA data addressed determinants of continuing labour participation in older workers. Work-related factors such as low physical workload and psychosocial support at work proved to be associated with continuation of paid work in workers with chronic diseases [138]. In a qualitative study in older workers with depression, cardiovascular disease and osteoarthritis, additional factors emerged as important for continuing work: sense of purpose in life, and—for depressed older workers—the life-structuring effect of work [139].

To facilitate the study of work demands in population-based studies of older workers, a general population job-exposure matrix (GPJEM) was developed [140]. Data on occupation-based work demands were derived from the Netherlands Working Conditions Survey. The GPJEM’s validity was supported by LASA data: physical demands were associated with self-rated health, functional limitations, and osteoarthritis of the hip and knee; psychosocial work demands in the absence of psychosocial support at work were associated with hypertension. Further research showed that workers in occupations with high cognitive demands more often reported memory complaints, independent of poor objective cognitive performance [141]. As memory complaints were found to predict future memory decline [142], a decrease in cognitive work demands may enable older workers to extend their working lives.

A longitudinal study of the effect of retirement on self-rated health in LASA-participants working at baseline showed that post-retirement self-rated health depended on the age at retirement [143]. Compared to participants who continued working, post-retirement self-rated health was worse in workers who retired at 55–58 and at 61–64 years, but was better in workers who retired at 59–60 years—the modal retirement age during the study period (1992–2006). In the latter group, financial incentives were likely to have led to early retirement, whereas health selection into early retirement was suggested to explain early exit in the other age groups.

Time spent on physical activity proved to increase after retirement [144]. The number of work-related personal ties decreased on average, but in the more recent generation (born 1938–1947) this decrease was significantly smaller than in the earlier generation (born 1928–1937) [145]. These findings suggest that retirement is inductive to positive health behaviour, and that retirement has become less socially disruptive in recent years.

### Care

With LASA data, the use of several types of care has been investigated: acute medical care (GP, medical specialists, hospital care), informal care by kin and non-kin, community care (home care provided by professionals or paid out of pocket) and institutionalized care.

Indicators of physical health, such as the presence of chronic diseases, the degree of functional limitations, and physical frailty, were all strongly related to the use of acute and long-term care in both cross-sectional and longitudinal studies [146–150]. In addition to physical health, indicators of cognitive decline and mental functioning were associated with higher likelihood of hospitalization [96, 151, 152], community services use [149, 151, 152] and residential care [132, 152, 153].

Individual dispositions to use community care were generally indicated by gender (women), higher age and lower level of education [146, 147]. Several psychological characteristics also impacted on the use of care: perceived control over care increased the likelihood of using privately paid care whereas lower levels of mastery increased the use of professional home care [154]. Positive psychological characteristics, related to sense of control, play a role in the transition between stages in the disablement process and lower the risk of institutionalization [155]. An ancillary study was conducted among LASA respondents to increase insight into how older adults conceptualize perceived control of care [156]. Based on these data, a measurement instrument to measure perceived control of care was developed and tested in the LASA sample [157].

The use of publicly provided care was generally negatively associated with the use of informal or privately paid care in LASA respondents. Social and economic resources, such as the presence of a spouse or other resident caregivers, children living in proximity, a large and diverse local social network, and income [1321, 146, 149] were important determinants of the use of informal care. A decline in social resources (living alone, lacking support) was associated with onset of residential care within a period of 3 years, but this association was not independent from detrimental developments of physical and cognitive functioning [158]. Data from another ancillary study showed that children were likely to complement the caregiving of their siblings [159].

Since 1992 the Dutch laws and systems for acute and long-term care have changed considerably resulting in a less generous and more means- and needs-tested system after the year 2007. Between 1992 and 2012 the receipt of informal care decreased considerably whereas the use of formal care increased slightly. Moreover, the negative associations between formal and informal care use decreased, suggesting a trend toward more complementarity between both forms of care after 2005 [160]. The cut-backs in professional care may have contributed to poor outcomes of care use: LASA respondents who received formal home care experienced more loneliness and less satisfaction with life in recent years, compared to those who received informal care or no care [161].

A few studies investigated the impact of trends in health and life expectancy on health service use and costs of care, using simulation studies based on latent Markov models [22, 162]. In these studies, multiple potential future health development scenarios were simulated to predict subsequent developments in the use of care and costs of care, including hospital care use as well as long-term care use.

### End of life

The end of life is studied in LASA by examining attitudes and preferences, wishes to die and end-of-life care. Additionally, in ancillary studies conducted in 2000 and 2010 close relatives of deceased participants were interviewed about the last phase of life of their deceased relative.

In 2008, 14 % of the participants indicated to want to live as long as possible, irrespective of health problems, while 86 % indicated to prefer a shorter life, if this could be without major health problems. Participants were also asked to specify their preference for receiving or forgoing four types of treatment: resuscitation, artificial nutrition and hydration, antibiotics, and artificial respiration, based on vignettes (a cancer and a dementia scenario). For all treatments and scenarios, a majority ranging between 57 and 84 % had a preference for forgoing such treatment [163]. Time trends analyses showed that the percentages of people with a positive attitude towards euthanasia and an end-of-life pill increased between 2001 and 2008 (from 58 to 70 % and from 31 to 45 %, respectively) [164]. The prevalence of possession of an euthanasia directive in LASA respondents was relatively stable around 6 % between 1998 and 2011 [165].

Actual treatments given in the last phase of life were compared with respondents` preferences for the four types of treatment mentioned earlier. People with a known preference for receiving a treatment had a seven times higher chance of their preference being followed than people with a known preference for forgoing that treatment [166, 167].

In 2005, 3.4 % of LASA participants reported currently having a wish to die and/or a weakened wish to continue living [168]. Additional qualitative interviews showed that a wish to die had either been triggered suddenly after traumatic life events or had developed gradually after a life full of adversity, as a consequence of aging or illness, or after recurring depression [169].

Care received at the end of life was compared between participants who deceased in 2000 and in 2010. In 2010, older people had more functional limitations three months before death. Over the 10-year period, people in the last phase of life were less likely to receive no care (12 vs 39 %) and more likely to receive formal home care (45 vs 15 %). People over 80 years, females, and those who died in 2010 were more likely to receive formal home and institutional care than informal or no care [170]. Finally, in 2010, 69 % of relatives reported that their relative had died with dignity [171], and a majority was satisfied with the communication between the physician, the patient and themselves [172].

## Data availability

LASA data are available for research. To obtain data, researchers need to submit an analysis proposal that is evaluated by the LASA Steering Group. The LASA Steering Group has adopted a policy of open sharing of data with interested researchers for specific research questions on aging-related issues. We invite colleagues to find out whether LASA data can help them to answer their research questions. More information on data requests can be found at the study website: www.lasa-vu.nl. Forms to request assessment of biomarkers are also available here. Data are available for investigation under the condition that results of analyses will be made available to the research community through scientific reports or research papers, regardless of the outcome of the study.
